# Development and physiology of GABAergic feedback excitation in parvalbumin expressing interneurons of the mouse basolateral amygdala

**DOI:** 10.14814/phy2.12664

**Published:** 2016-01-05

**Authors:** Jay Spampanato, Robert K. P. Sullivan, Madhusoothanan B. Perumal, Pankaj Sah

**Affiliations:** ^1^ The Queensland Brain InstituteThe University of QueenslandSt. LuciaAustralia

**Keywords:** Axoaxonic, axon initial segment, chandelier neuron, network

## Abstract

We have previously shown that in the basolateral amygdala (BLA), action potentials in one type of parvalbumin (PV)‐expressing GABAergic interneuron can evoke a disynaptic feedback excitatory postsynaptic potential (fbEPSP) onto the same presynaptic interneuron. Here, using whole‐cell recordings from PV‐expressing interneurons in acute brain slices we expand on this finding to show that this response is first detectable at 2‐week postnatal, and is most prevalent in animals beyond 3 weeks of age (>P21). This circuit has a very high fidelity, and single action potential evoked fbEPSPs display few failures. Reconstruction of filled neurons, and electron microscopy show that interneurons that receive feedback excitation make symmetrical synapses on both the axon initial segments (AIS), as well as the soma and proximal dendrites of local pyramidal neurons, suggesting fbEPSP interneurons are morphologically distinct from the highly specialized chandelier neurons that selectively target the axon initial segment of pyramidal neurons. Single PV interneurons could trigger very large (~ 1 nA) feedback excitatory postsynaptic currents (fbEPSCs) suggesting that these neurons are heavily reciprocally connected to local glutamatergic principal cells. We conclude that in the BLA, a subpopulation of PV interneurons forms a distinct neural circuit in which a single action potential can recruit multiple pyramidal neurons to discharge near simultaneously and feed back onto the presynaptic interneuron.

## Introduction

Neocortical areas of the mammalian central nervous system contain two main types of neuron: glutamatergic principal (or pyramidal) neurons, and GABAergic interneurons. In mature cortical regions, principal neurons make local as well as long‐range excitatory connections, whereas GABAergic neurons generally make local circuit inhibitory connections, although some long‐range projections have also been described (Jinno et al. 2007; Klausberger and Somogyi 2008; Mascagni and McDonald 2009). GABAergic interneurons are divided into a number of distinct families based on their morphology, cellular markers and axonal targets (Markram et al. 2004; Ascoli et al. 2008; Klausberger and Somogyi 2008). Of these, the axo‐axonic cells (AACs) are thought to exclusively innervate the axon initial segment (AIS) of principal neurons (Howard et al. 2005). AACs express the calcium binding protein parvalbumin (PV), and form cartridge‐type synapses with 5–7 boutons along the AIS (Howard et al. 2005; Klausberger and Somogyi 2008; Taniguchi et al. 2013). Due to their distinct axonal morphology in laminar cortical areas, these interneurons have also been called chandelier cells. The basolateral amygdala (BLA) is a cortical‐like structure, and as such contains a similar distribution of interneuron types as that seen in the cortex and hippocampus (Spampanato et al. 2011). Thus, PV‐expressing interneurons are present in the BLA (Pare and Smith 1993; Woodruff and Sah 2007b; McDonald et al. 2012), and PV‐positive chandelier‐type cartridge synapses have also been described (McDonald and Betette 2001; Muller et al. 2006; Bienvenu et al. 2012).

At synapses made by GABAergic interneurons, the neurotransmitter GABA activates postsynaptic ionotropic receptors that form anion‐selective ligand‐gated ion channels with primary permeability to chloride ions (Hille 2001). In neurons, cytosolic chloride is regulated by the activity of a family of cation chloride cotransporters (Blaesse et al. 2009). Of these, the Na‐K‐2Cl importer, NKCC1, transports chloride ions into neurons using the Na^+^ gradient, while the KCl exporter, KCC2, transports chloride ions out of neurons using the K^+^ gradient. These transporters are developmentally regulated, and in mice, NKCC1 is highly expressed in the plasma membrane of most neurons up to postnatal day five (P5), but is subsequently downregulated (Payne et al. 2003). In contrast, KCC2 expression begins around P6, and is then maintained throughout life (Rivera et al. 2005). Thus, early in development, NKCC1 activity maintains a high intracellular chloride concentration, and at resting membrane potentials, GABAergic synapses are depolarizing (Payne et al. 2003; Blaesse et al. 2009). With development, increasing expression and activity of KCC2, coupled with down regulation of NKCC1, reduces cytosolic chloride, and in mature neurons GABAergic synapses are inhibitory (Payne et al. 2003; Blaesse et al. 2009).

As AACs innervate the AIS, they were originally thought to provide a powerful source of inhibition directly onto the site of action potential initiation (Kole and Stuart 2012). However, recent studies have shown that synapses made by AACs in the cortex can be excitatory, and have been suggested to even trigger action potentials in the postsynaptic cell (Szabadics et al. 2006; Molnar et al. 2008; Woodruff et al. 2009). Moreover, in some cases, principal neurons receiving excitatory AAC synapses have been found to reciprocally innervate the AAC, resulting in an excitatory feedback loop back onto the AAC (Szabadics et al. 2006; Woodruff et al. 2006; Molnar et al. 2008). This depolarizing GABAergic response at the AIS of mature cortical pyramidal neurons has been suggested to result from a depolarizing chloride gradient at the AIS maintained by a combination of a lack of KCC2 expression and continued activity of NKCC1 (Szabadics et al. 2006; Khirug et al. 2008).

Interneurons that selectively make axo‐axonic synapses have been described in the BLA (Bienvenu et al. 2012; Veres et al. 2014), and in mature animals, paired recordings have demonstrated that these connections are inhibitory (Veres et al. 2014). In contrast, we have shown that in one type of PV‐expressing interneuron of the BLA, an action potential results in generation of a time‐locked ‘feedback’ EPSP (fbEPSP) onto the same presynaptic interneuron (Woodruff et al. 2006). This feedback EPSP is similar to that described for AACs in the cortex (Szabadics et al. 2006; Molnar et al. 2008), suggesting that some PV interneurons in the adult BLA make excitatory connections. However, whether these interneurons were AAC type neurons was not confirmed. Here, we describe the developmental time course of this feedback circuit in the BLA, and its physiological properties. We show that interneurons that display feedback EPSPs in the BLA form synapses with three distinct postsynaptic regions: dendrite, soma and AIS. Thus, cells with feedback excitation share properties with basket‐cells, rather than the AACs that have been recently described in the BLA (Bienvenu et al. 2012; Veres et al. 2014). We show that discharge of a single PV interneuron in mouse BLA can produce large synchronized fbEPSCs as well as complex time‐locked polysynaptic events. Together, these results describe a novel interneuron circuit in the BLA.

## Materials and Methods

All procedures were conducted in accordance with the Australian Code of Practice for the Care and Use of Animals for Scientific Purposes, and were approved by the University of Queensland Animal Ethics Committee. Heterozygous transgenic mice expressing enhanced green fluorescent protein (EGFP) under control of the parvalbumin (PV) promoter were bred and maintained on the BALB/c background as previously described (Woodruff et al. 2006).

### Electrophysiology

Male and female mice aged 9–44 days were anesthetized through ambient inhalation of isoflurane prior to decapitation. Brains were removed rapidly and transferred to ice‐cold choline chloride artificial cerebrospinal fluid (aCSF) containing (in mmol/L): 118 C_5_H_14_ClNO, 2.5 KCl, 2.5 CaCl_2_, 1.3 MgCl_2_, 1.2 NaH_2_PO_4_, 10 Glucose, and 25 NaHCO_3_, bubbled with carbogen (95% O_2_, 5% CO_2_) to yield pH 7.3–7.4. Coronal sections containing BLA were cut, 350 *μ*m thick, using a Leica VT‐1000S Vibratome (Leica Microsystems, Wetzlar, Germany), and transferred to normal aCSF (choline chloride replaced with equimolar NaCl) at 32–34°C. Slices were incubated for at least 1 h prior to experimentation.

Slices were individually placed into a submerged recording chamber and perfused with carbogen gassed aCSF maintained at 34 ± 2°C (TC‐324B, Warner Instruments, LLC, Hamden, Connecticut). Microelectrodes (3–6 MΩ) were pulled from borosilicate glass and filled with an internal recording solution containing (in mmol/L): 135 KMeSO_4_, 5 NaCl, 10 HEPES, 2 Mg_2_‐ATP, 0.3 Na_3_‐GTP, 0.3 EGTA, 0.1 spermine, 7 phosphocreatine, and 8 biocytin (pH = 7.3, ~290 mOsmol). Whole‐cell recordings were obtained from visually identified EGFP‐expressing PV interneurons in the BLA using an upright microscope (Olympus BX50WI, Olympus Optical, Tokyo, Japan) equipped with a fluorescence attachment. Data were collected using Axograph X software (Axograph Scientific, Sydney, Australia) using a Multiclamp 700B amplifier (Molecular Devices, Sunnyvale, CA). Signals were filtered at 10 kHz and digitized at 50 kHz using an ITC‐16 A/D converter (InstruTECH, HEKA, Ludwigshafen/Rhein, Germany).

PV‐EGFP interneurons were patched at random throughout the BLA. Each patched cell was determined to have a feedback excitatory postsynaptic current or potential (fbEPSC/P) using a protocol (voltage clamp) in which a 1–1.5 msec square depolarizing pulse to 0 mV was delivered from a holding potential of −70 mV at 0.33 Hz. In current clamp a similar short current injection evoked an action potential under similar conditions. If no time‐locked excitatory response could be observed following the depolarization the cell was defined as a nonfeedback cell. The presence of the fbEPSC was confirmed by recognition of a time‐locked event during subsequent calculation of a cumulative probability and event frequency histogram for each cell. Events were detected using the Axograph X software template matching function. The individual template was adjusted on a cell‐by‐cell basis when necessary to assure that nearly 100% of the events were detected in each sweep for each cell. Pharmacological reagents were dissolved in water when possible or DMSO when necessary. Drugs dissolved in DMSO were added to the recording solution from a 1000‐fold stock and the concentration of DMSO (0.001%) was maintained constant throughout the experiments. Each drug was perfused into the bath for at least ten minutes prior to the start of drug‐on protocols and the fbEPSP was monitored during the drug application at a rate of 0.1 Hz. All average data are presented as mean ± 1 standard deviation (SD). Statistical analyses were performed using SigmaPlot 12.5 (Systat Software Inc., Chicago, IL).

### Immunohistochemistry

For neurons filled with biocytin, slices were fixed overnight at 4°C in 4% paraformaldehyde with 0.5% glutaraldehyde in 0.1 mol/L phosphate buffered saline (PBS), washed in PBS, embedded in 4% agarose and subsectioned at 50 *μ*m using a Leica VT‐1000S Vibratome. Sections were incubated in 1% H_2_0_2_ for 30 min and then washed in 0.1 mol/L PBS. Sections were blocked in 3% BSA, 0.05% saponin and then incubated overnight in avidin‐conjugated HRP (1:5000) in blocking solution (0.5% BSA, 0.05% saponin, 0.05% thimerosal in 0.1 mol/L PBS) and developed with nickel‐DAB. Following recovery, sections were incubated first in sodium azide to inactivate any remaining HRP activity and then incubated in anti‐Ankyrin‐G (Rabbit, 1:500, Santa Cruz) in blocking solution for ~56 h at room temperature, followed by an incubation in biotinylated anti‐rabbit secondary antibodies (1:500, Jackson Biosciences) for 5 h and then avidin‐conjugated HRP overnight. Immunolabelling was revealed using either DAB or VIP (Vector) as an alternative color to the filled cell. Alternatively biocytin‐filled cells were recovered using streptavidin Alexa‐555 (1:2000, Invitrogen) for fluorescence microscopy. Fluorescence immunolabelling was also performed (on tissues P35‐P42, or younger tissue where indicated) using antibodies to KCC2 (R‐14, goat, 1:500, Santa Cruz), NKCC‐1 (N‐16, goat 1:1000, Santa Cruz) and Parvalbumin (P3088, mouse, 1:2000, Sigma‐Aldrich) and detected using species‐specific fluorophore‐conjugated secondary antibodies (Alexa‐488, ‐568 and ‐647, Invitrogen). Labeled sections were mounted onto slides in 50% glycerol/PBS and images taken using Axioimager (Zeiss). Imaged sections were used to generate schematic drawings of biocytin‐filled cells relative to Ankyrin‐G labeled AIS to identify putative synaptic connections onto AIS and somas. These schematic drawings were used as a guide to correlate light and electron microscopy observations.

### Electron microscopy

Imaged sections were postfixed in 1% OsO_4_ in 0.1 mol/L sodium cacodylate buffer at 80 W in a Biowave microwave using a 2 min on 2 min off cycle under vacuum. Sections were flattened and then dehydrated in a graded series of acetone (50%, 70%, 90%, 95%, 2 × 100%) at 250 W without vacuum for 40 sec each. Infiltration of Epon resin was performed using graded series of Epon in acetone (25%, 50%, 75%, and 2 × 100%) at 250 W for 3 min with vacuum. Tissues were placed into fresh resin and polymerization at 60°C for 48 h. Sixty nanometer thick sections were cut using a Leica ultramicrotome and collected onto grids before being contrast‐enhanced with uranyl acetate and lead citrate and then viewed using a JEOL 1010 transmission electron microscope. Digital images were captured on a Morada CCD camera. Photomicrographs were viewed and plates created with Photoshop and Illustrator CS3 (Adobe Systems Incorporated, San Jose). Minor adjustments were made to brightness and contrast of images.

## Results

### Development and fidelity of the feedback circuit

**Figure 1:**
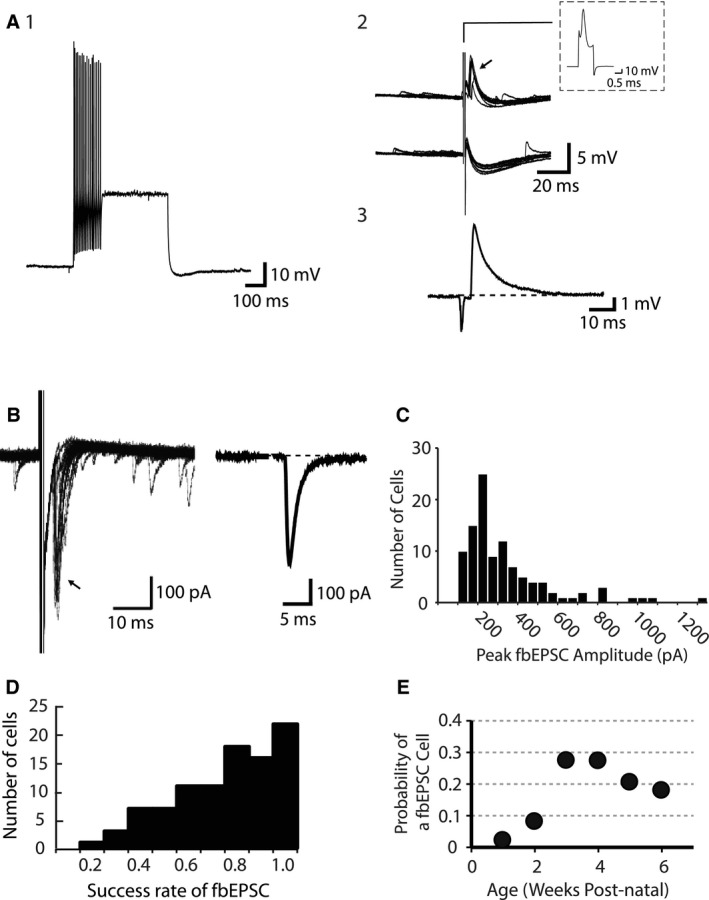
Development and Fidelity of Feedback Cells in the BLA. (A) PV‐EGFP interneurons in the basolateral amygdala discharge at high frequency and single action potentials can be followed by a feedback excitatory postsynaptic potential (fbEPSP). A1, Shown is the response of a PV‐EGFP neuron to twice threshold current injection. The response to a single action potential is shown at higher gain in A2. Upper traces show instances where an action potential is followed by a fbEPSP (arrow) whereas instances in which there was failure to evoke a fbEPSP are shown in the lower traces. The action potential (truncated in traces on the left) evoked by a current injection is shown in the inset. A3, Subtraction of the average of traces with failures from those with successes reveals the fbEPSP in isolation. (B) In voltage clamp (V_h_: −70 mV), a brief (1 msec) depolarizing voltage step to 0 mV evokes a fbEPSC – 10 successful traces have been superimposed (arrow). Subtraction of the unsuccessful voltage steps shows the fbEPSC (right). (C) The histogram plots the amplitude of the fbEPSC for each cell (*n* = 96). (D) Fidelity histogram demonstrating the probability of evoking a fbEPSP in single cells. The success rate was calculated as the proportion of trials in which a fbEPSP was evoked in a series of 40 trials at 0.33 Hz. (E) The probability of a recorded cell having a fbEPSC was calculated for animals from at least 1 week to at least 6 weeks of development. The data represent the number of fbEPSC cells divided by all the EGFP cells patched for that age range. 1 week (p7–p13): 5 animals, 1/45 cells. 2 weeks (p14–p20): 5 animals, 4/49 cells. 3 weeks (p21–p27): 18 animals, 39/142 cells. 4 weeks (p28–p34): 14 animals, 29/106 cells. 5 weeks (p35–p41): 9 animals, 14/68 cells. 6 weeks (p42–p48): 5 animals, 9/50 cells.

Whole‐cell recordings were made from PV‐interneurons in the BLA of mice‐expressing EGFP under control of the PV promoter (Meyer et al. 2002). These interneurons express PV (Meyer et al. 2002; Rainnie et al. 2006; Woodruff et al. 2006; Woodruff and Sah 2007b), and have been classified into four groups based on their firing properties, and local connectivity (Rainnie et al. 2006; Woodruff and Sah 2007b). As previously shown (Woodruff et al. 2006), in one class of PV‐interneuron, a single action potential evoked a delayed feedback excitatory postsynaptic potential (fbEPSP) that appears superimposed on the after hyperpolarization that follows the action potential (Fig. 1A). Subtraction of episodes where no fbEPSP was evident from episodes where it is present (Fig. 1A2) reveals the fbEPSP (Fig. 1A3). With neurons voltage clamped at −70 mV, a brief (1–1.5 msec) voltage step to 0 mV results in an unclamped “action current” that evoked a time‐locked feedback excitatory postsynaptic current (fbEPSC) (Fig. 1B) that underlies the fbEPSP (Woodruff et al. 2006). The amplitude of the fbEPSC was broadly distributed (Fig. 1C) with a mean of 290 ± 223 pA (median: 211 pA; *n* = 96). The fbEPSC was not detected following every action potential (Fig. 1A2), however, in any one neuron the failure rate was low, with a mean within‐cell success rate of evoking the fbEPSC of 0.72 ± 0.22 (Fig. 1D). That is, on average, an action potential in the PV‐interneuron triggered a time‐locked fbEPSC approximately 72% of the time. Of the 96 feedback cells recorded, 56 cells (58% of the total) maintained a success rate greater than 0.7, similar to that observed in cortical AACs (Szabadics et al. 2006), and 22 cells (23% of the total) had a success rate >0.9. When continuous spike trains were elicited by somatic current injection, these fbEPSC neurons displayed either fast‐spiking or stuttering phenotypes (Woodruff et al. 2006). In the current sample, from 32 neurons with feedback connections, in which firing properties were clearly established, 20 (62.5%) were stutterers and the rest were fast‐spiking neurons.

PV‐interneurons that have fbEPSCs are GABAergic (Meyer et al. 2002; Woodruff et al. 2006; Woodruff and Sah 2007b), and as expected, the fbEPSC was blocked by the GABA receptor antagonists picrotoxin (*n* = 7), SR95531 (*n* = 4), and bicuculline (*n* = 12) (Fig. 5). However, the fbEPSC is also blocked by the AMPA‐kainate receptor antagonist CNQX (Woodruff et al. 2006). It has therefore been proposed that the fbEPSC results from activation of a disynaptic circuit in which GABA released at terminals of these interneurons is depolarizing, driving a postsynaptic glutamatergic neuron to threshold that then makes synapses back onto the interneuron (Szabadics et al. 2006; Woodruff et al. 2006; Molnar et al. 2008). All neurons in this, and our previous study (Woodruff et al. 2006) were identified in PV‐EGFP mice, where EGFP expression was driven from the PV promoter using a BAC (Meyer et al. 2002). We were therefore concerned that the feedback EPSC may result from off target physiological effects of this genetic manipulation. Therefore, recordings were also made from interneurons using GAD67‐EGFP mice (Tamamaki et al. 2003). Feedback cells with identical properties were readily found in these mice and were positive for PV (data not shown).

In rodent cortical neurons, GABA is typically inhibitory from about the second postnatal week due to increasing expression of KCC2 (Rivera et al. 1999, 2005; Tyzio et al. 2007). How is it then possible that a population of PV‐interneurons in mature animals is excitatory? The developmental time course of intracellular chloride concentration can differ significantly within the cortex, and between different brain regions (Glykys et al. 2009). To date, studies showing excitatory actions of GABA have used slices from young animals (Szabadics et al. 2006; Woodruff et al. 2006, 2009, 2011; Khirug et al. 2008). Thus, one possibility is that the presence of excitatory GABAergic synapses may simply reflect a late switch from the embryonic high intracellular chloride in a compartment of some principal neurons. We therefore investigated the developmental time course of the fbEPSC over the age range P9 to P44. Animals were grouped by completed weeks of postnatal development such that an animal in its second week of life has completed one full week of development and is therefore 1‐week old: day age range P7–P13. Whole‐cell recordings were made from PV‐EGFP interneurons in the BLA, and each cell was tested for the presence of a feedback postsynaptic current/potential as shown in Figure 1B. The probability of finding a feedback interneuron in slices from animals for each age range is shown in Figure 1E. Relative to the timing of the developmental switch from excitatory to inhibitory GABA in the cortex, hippocampus and amygdala (Rivera et al. 1999, 2005; Tyzio et al. 2007; Ehrlich et al. 2013), the appearance of fbEPSCs occured later in development with onset beyond 2 weeks of age. Thus, interneurons with fbEPSCs were most prevalent in 3‐week‐old animals (39 out of 142 cells: 27%; 18 animals P21–P27) and remained so in more mature animals (52 out of 224 cells: 23%; 28 animals P28–P44). In slices prepared from animals less than 3‐week old, interneurons displaying fbEPSC were relatively rare (5 of 94 cells: 5%; 10 animals P9–P20), with the earliest occurrence of a feedback cell found at P12 (1 of 45 cells, with P9 being the earliest age investigated).

We found that the age of the animal was a significant factor in the probability of finding a feedback cell in the BLA (ANOVA on Ranks, *P* < 0.01). The probability of finding a feedback cell in animals 20 days old or younger was significantly lower than in animals P21 or older (Mann–Whitney *U*‐test, *P* < 0.01). In contrast, there was no correlation between the age of the animal and the success rate of the fbEPSC within a given cell (*P* > 0.05, one‐way ANOVA). The within‐cell success rates of the fbEPSC by age were (average ± SD): 1 week: 0.93 (no SD, *n* = 1), 2 weeks: 0.64 ± 0.18, 3 weeks: 0.76 ± 0.20, 4 weeks: 0.76 ± 0.19, 5 weeks: 0.63 ± 0.25, 6 weeks: 0.62 ± 0.26. These success rates are similar to the success rate reported for GABAergic PV‐interneurons that have also been demonstrated to trigger disynaptic EPSPs in the cortex (Szabadics et al. 2006; Molnar et al. 2008).

In the cortex, AACs form a developmentally and morphologically distinct population of interneurons that selectively innervate the AIS of local principal neurons (Somogyi et al. 1982; Howard et al. 2005; Taniguchi et al. 2013). Moreover, some AACs can also receive feedback excitation, similar to that seen in the BLA (Szabadics et al. 2006; Molnar et al. 2008). Two mechanisms have been suggested to account for the depolarizing action of GABA at the AIS. One study suggested that KCC2 expression in the AIS is low, and as a result, the AIS maintains a depolarizing chloride gradient (Szabadics et al. 2006). Alternatively, a different study has suggested that unlike the soma and dendrites, expression of NKCC1 at the AIS is high, raising local cytosolic chloride in the AIS, again resulting in a depolarizing chloride reversal potential at AIS targeting synapses (Khirug et al. 2008). We therefore tested if BLA interneurons that display fbEPSCs may also synapse on the AIS of local pyramidal neurons.

**Figure 2:**
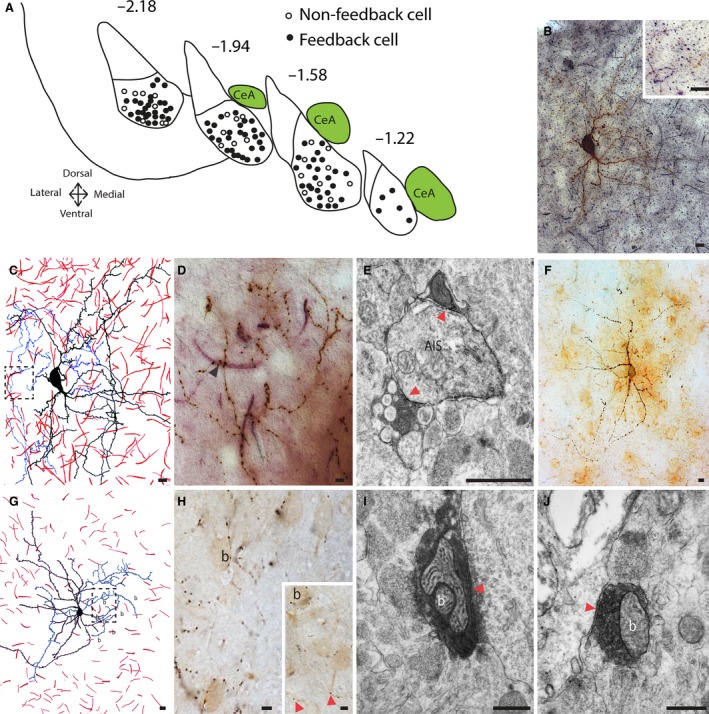
Feedback cells make synaptic connections onto axon initial segments as well as soma and dendrites. (A) Distribution of recovered biocytin‐filled feedback (closed circle, *n* = 83) and nonfeedback (open circle, *n* = 25) parvalbumin‐EGFP positive cells in the BLA at −2.18, −1.94, −1.58, −1.22 from bregma. (B) A recovered feedback cell filled with biocytin (brown) and colabeled with Ankyrin‐G (black or pink) were examined at the light microscopy level (B, C, D) to identify putative synaptic contacts. The axon of a recovered feedback cell made numerous single or multiple putative contacts (arrowheads in high magnification inset and (D) onto surrounding AIS and dendrites. (C) Schematic drawing corresponds to the biocytin‐filled cell in B with the dendrites in black, axon in blue and AIS in red. Putative synaptic connections identified in B–D were verified using electron microscopy. (E) DAB precipitates evident in the labeled axons made synaptic connections (red arrowheads) onto AIS. (F) Different recovered feedback cell filled with biocytin. G, H, Neuron in (F) is drawn with the dendrites in black and axon in blue. Putative basket synapses are labeled (B), and shown on an expanded scale in the inset. I, J, two putative contacts from the filled axon (G, red arrowheads) are shown making symmetrical synapses on the soma of nearby neurons. Scale bars in A, B, C, F, G, H = 10 *μ*m; E, I, J = 1 *μ*m.

Electrophysiologically identified feedback and nonfeedback cells were distributed throughout the BLA (Fig. 2A). To determine the synaptic targets of these interneurons, cells were filled with biocytin and slices were colabeled for the AIS marker Ankyrin‐G (Fig. 2B, D). The axon, dendrites, and soma of each recovered feedback cell were drawn and all putative axo‐axonic and basket‐type somatic synapses counted (Fig. 2C, G). Putative contacts onto the AIS of neighboring neurons were observed for all feedback cells (*n* = 15 filled neurons) with a mean of 6.5 AIS contacted per neuron, range 1–20 AIS contacted. Of these, three of the recovered feedback cells exhibited cartridge‐like synapses with at least three putative contacts evident on a single Ankyrin‐G‐labeled AIS (mean: 3.7 contacts/AIS). These putative contacts onto the AIS were confirmed to be symmetric, presumably GABAergic, synapses using electron microscopy. DAB‐electron dense precipitates present in the axon of the recovered feedback cells made synapses on to AIS (*n* = 4; Fig. 2E). In 10 of these 15 feedback cells, putative basket‐type somatic synapses were also observed (Fig. 2F–J) with a mean of 3.9 basket synapses per cell (range 1–21). In total, 57 putative synapses from four filled feedback cells were identified as symmetrical synapses on electron microscopic analysis. Of these, 39 contacts (68.5%) were on dendrites, 11 (19.5%) were on the AIS, and 7 (12%) were somatic. Thus, PV‐positive cells that initiate feedback EPSCs in the BLA make AIS synapses, but also innervate other neuronal regions, including other parvalbumin‐expressing interneurons (Woodruff et al. 2006). Similar to AIS synapses, somatic contacts (Fig. 2H) were confirmed to be symmetric, presumably GABAergic, synapses using electron microscopy (*n* = 7; Fig. 2I, J).

**Figure 3:**
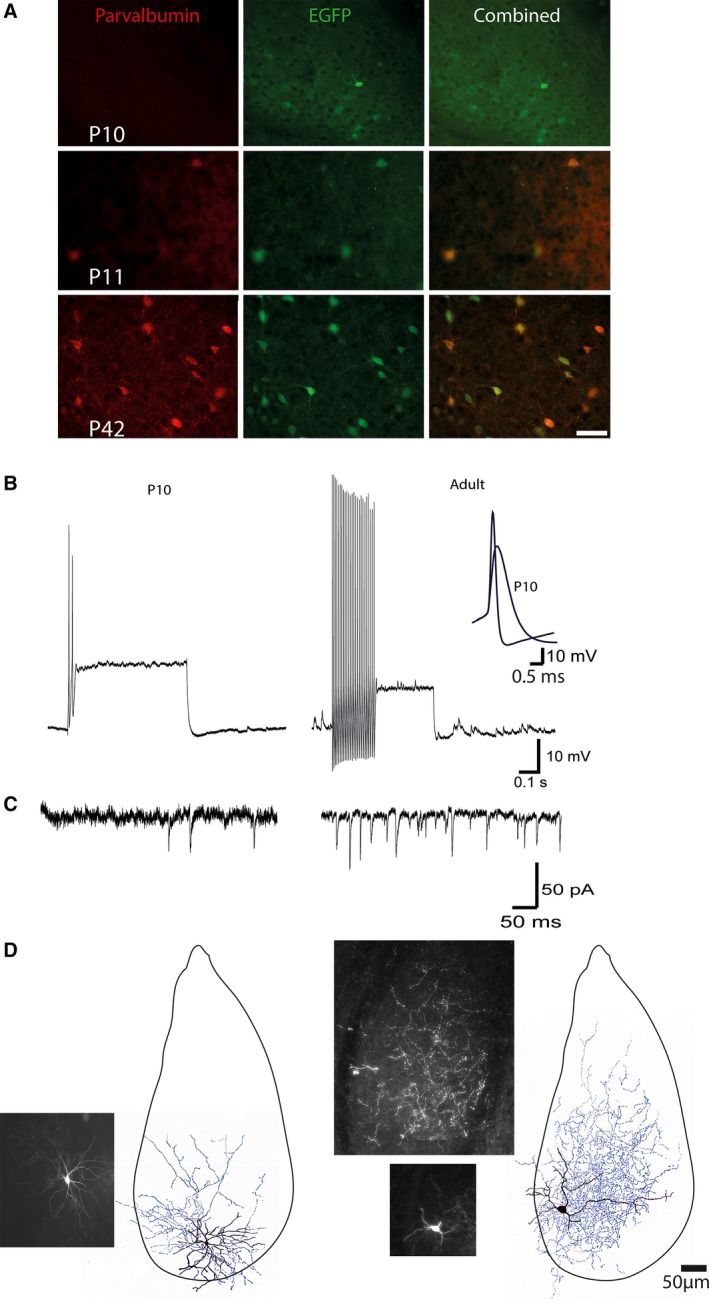
Development of parvalbumin interneurons. Co‐localization of parvalbumin and EGFP in cells within the BLA of PV‐EGFP mice at P10, P11, and P42. A. Parvalbumin was not detectable at P10, although EGFP was present in a few cells. Parvalbumin expression was present in EGFP positive cells at P11. In older animals (P42), parvalbumin was evident in all EGFP+ cells. B. Immature (P10) EGFP+ cells fired only a few action potentials in response to suprathreshold current injections while older EGFP+ cells (>P21)fired at high frequencies typical of PV+ interneurons. The immature cells also had single action potential waveforms that were smaller and slower (inset). (C) Immature EGFP+ cells displayed excitatory postsynaptic currents at lower frequency than in the older animals (>P21). (D) The cells in B were filled with biocytin and recovered. Fluorescent images of each cell are shown with an additional, deeper image, for the mature cell demonstrating the extensive length of the axon. A full anatomical reconstruction of each cell is shown with the cell body and dendrites in black and axon in blue to demonstrate that the EGFP+ cells in immature animals were also physically smaller and had a smaller axonal arbor than in the older animals (>P21).

In interneurons, PV expression using standard immunohistochemistry is not detectable till P10‐P12 (del Rio et al. 1994; Berdel and Morys 2000), however, in PV‐EGFP mice EGFP expression was clearly detectable by P7. As these mice were generated using a BAC, it is possible that the promoter is lacking regulatory elements, or that these elements are overridden by the positional effects related to the location of the insertion as often happens in mouse transgenic lines (Feng et al. 2000). Alternatively expression of EGFP could simply be higher than that of parvalbumin at these ages allowing for the visual identification of green light at a higher sensitivity than the antibody reaction for parvalbumin. Importantly, whenever PV could be detected by antibody reaction, EGFP positive cells were always parvalbumin positive (Fig. 3A) (Meyer et al. 2002; Woodruff et al. 2006). We are therefore confident that EGFP expressing neurons are indeed PV‐positive interneurons. At P10/11 when fbEPSCs were not detected, EGFP positive neurons had resting potentials of −77 ± 14 mV and depolarizing current injection evoked action potentials that were smaller in amplitude and significantly broader in half width than those in older animals (>P21) (1.1 ± 0.2 msec in P10/11 compared with 0.60 ± 0.01 msec in older neurons; Fig. 3B). The frequency of spontaneous excitatory synaptic currents at P10/P11 (6.9 ± 1.1 Hz; *n* = 3) was consistently lower than that seen in more mature neurons (88 ± 37 Hz, *n* = 3; Fig. 3C). Moreover, the axonal arborization of these neurons was clearly less extensive than in the older neurons (Fig. 3D). Thus, in agreement with a previous study (Ehrlich et al. 2012), these data show that as with pyramidal neurons (Ehrlich et al. 2013), PV+ interneurons are still maturing at ~P10, and the inability to detect fbEPSPs most likely reflects the immaturity of synaptic connections at this age.

### Synchronization and polysynaptic feedback circuits

**Figure 4:**
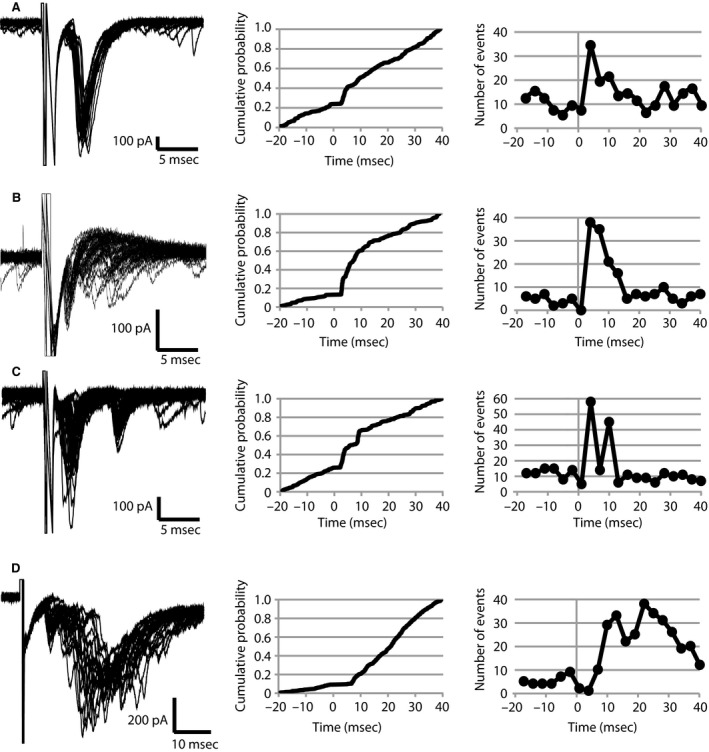
Temporal Precision of Polysynaptic fbEPSC Circuits. (A) *Left:* Raw traces (black) representing successful trials demonstrating a large amplitude fbEPSC. The fbEPSC follows a 1 msec step depolarization to 0 mV. The capacitive artifact and escape current can be seen prior to the fbEPSC. Spontaneous background activity can also be seen in the raw traces. *Center:* The cumulative probability plot confirms the precision of the time‐locked event. The voltage step is set to time 0 and corresponds to the flat section where no events could be detected. The vertical deflection of the line demonstrates an increased probability of an event occurring at that time. *Right:* The peak of the frequency histogram occurs during the 1–4 msec bin. (B) *Left:* Raw traces from a feedback cell with a highly precise primary fbEPSC followed by an asynchronous secondary fbEPSC. *Center/Right:* Cumulative probability plot shows a near vertical increase in probability at 3 msec followed by above background activity from 3 to 10 msec. The asynchronous activity can be seen as a broad peak on the frequency histogram. (C) *Left:* Raw traces from a feedback cell with two time‐locked fbEPSCs. The secondary fbEPSC rarely occurred independent of the primary fbEPSC. *Center/Right:* The precision of the secondary fbEPSC can be seen as a second step in the cumulative probability plot and a second peak in the frequency histogram. (D) *Left:* Raw traces demonstrating complex poly‐synaptic fbEPSCs following a late time‐locked primary fbEPSC. *Center/Right:* The cumulative probability plot and frequency histograms demonstrate the sustained increase in EPSCs on to the feedback cell following a single depolarization.

As described previously (Woodruff et al. 2006), in most neurons the fbEPSC had a latency of 2–5 msec following the action potential, displayed considerable latency jitter, and had peak amplitudes <400 pA (Fig. 1C). However, in this much larger cohort of cells, we found several variations of these properties (Fig. 4). Seven cells received large amplitude fbEPSCs (>600 pA; *V*_h_ = −70 mV), with the largest being over 1000 pA (e.g., Fig. 4A). These cells were found in animals aged P21 (*n* = 3), P23, P24, P34, and P42. The latency of these large amplitude fbEPSCs was similar to that seen for smaller amplitude events, as can be seen in the cumulative probability and frequency histograms (Fig. 4A). Moreover, in ten cells (animals aged P12, P21 (*n* = 2), P22, P23, P24, P25, P28, P29, P34), multiple fbEPSCs were seen with one primary time‐locked fbEPSC followed by one or more secondary fbEPSCs (Fig. 4B). This can be seen in the raw data, as well as in the cumulative probability plot, which demonstrates a highly precise primary fbEPSCs in the form of a steep vertical slope at 3–4 msec followed by a higher than background level of activity in the 5–10 msec period (Fig. 4B). These secondary events did not require the presence of a short‐latency primary event, and could be either due to a polysynaptic circuit or delayed transmitter release from multiple synapses. In four other cells (animals aged P23, P27, P28, P42), the secondary fbEPSCs were also time‐locked (Fig. 4C), and this fixed temporal nature of the secondary EPSCs can be seen as a second “step” in the cumulative probability and a second peak in the frequency histogram, suggesting the presence of a highly precise polysynaptic circuit. Last, in three cells (animals aged P23, P35, P36) there was an initial early time‐locked event that was followed by a barrage of secondary fbEPSCs that lasted many tens of milliseconds (Fig. 4D). In each case, the phenotype observed in voltage clamp mode was confirmed in current clamp to eliminate issues that might arise regarding the artificial triggering of an action current by a square voltage step.

### Mechanism of feedback excitation

**Figure 5:**
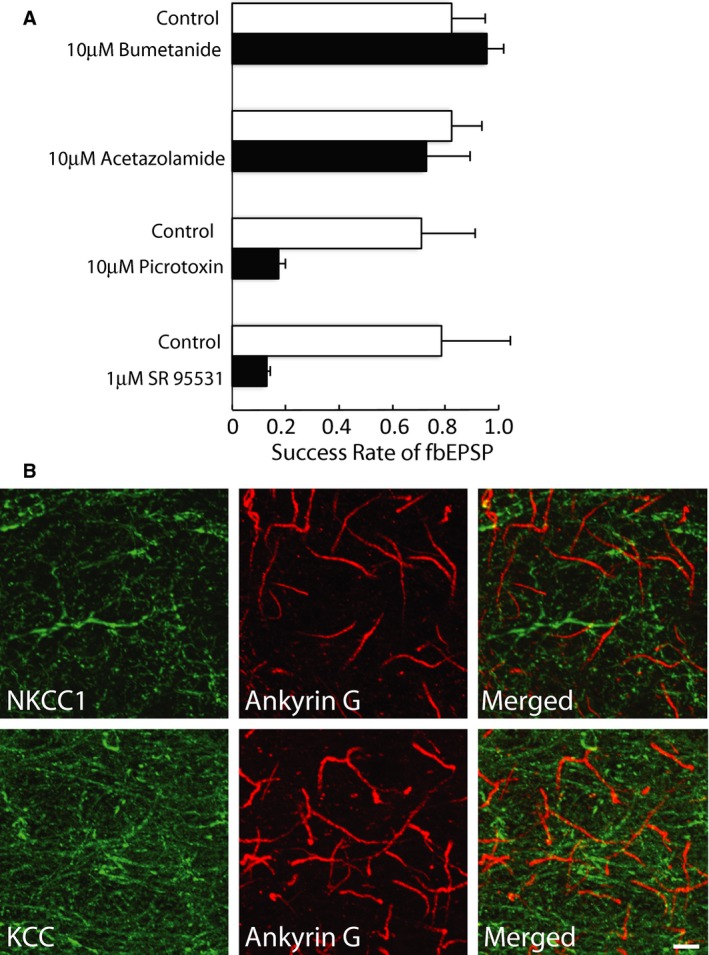
Feedback EPSP does not require NKCC1 activity. (A) Blocking NKCC1 (bumetanide, 10 μmol/L) or carbonic anhydrase (Acetazolamide) had no effect on the success rate of evoking the fbEPSC. The fbEPSP is blocked by blocking GABAA receptors with 1 μmol/L SR95531 (or 10 μmol/L picrotoxin). (B) Chloride transporters NKCC1 and KCC2 are targeted away from the AIS of principal cells in the BLA. Images show dual‐immunofluorescence labeling for Ankyrin‐G (red) and the chloride transporters NKCC1 or KCC2 (green). Merged images exhibited no co‐expression of chloride transporters with Ankyrin‐G. Scale bar = 10 *μ*m.

We have shown that in the BLA, action potentials in one type of PV‐expressing interneuron generate a feedback EPSP that follows the action potential. This feedback EPSP has a long latency (>3 msec), shows significant onset jitter, and is blocked by both GABAergic and glutamatergic antagonists. The most parsimonious explanation of this data, and one previously reached for AACs in the cortex, is that fbEPSPs result from an excitatory action of GABA at some principal neurons that in turn innervate the presynaptic interneuron (Szabadics et al. 2006; Woodruff et al. 2006; Molnar et al. 2008). As described above, antagonizing GABA_A_ receptors with picrotoxin (100 μmol/L, *n* = 7, *P* < 0.05, paired *t*‐test), SR95531 (1 μmol/L, *n* = 4, *P* < 0.05, paired *t*‐test), or bicuculline (10 μmol/L; *n* = 12, *P* < 0.05, paired *t*‐test) blocked the fbEPSP (Fig. 5A). What then explains the excitatory nature of these AIS synapses in the older animals?

To test if the depolarizing action of GABA in the BLA was due to NKCC1 expression in the AIS, we determined the effect of the NKCC1 antagonist bumetanide. To avoid disruption of the ionic gradients of the glutamatergic postsynaptic cell, we measured the output of the circuit, the fbEPSP success rate. Bumetanide had no effect on the fbEPSP (*n* = 4; Fig. 5A). Thus, the depolarizing action of GABA is unlikely to result from accumulation of chloride in the AIS due to activity of NKCC1. Consistent with this data, immunohistochemical staining for NKCC1 failed to label this transporter on the AIS in the BLA (Fig. 5B). The potassium‐chloride transporter KCC2 was expressed in dendrite‐like processes but was also not detectable at the AIS of principal cells in the BLA (Fig. 5B). Positive and negative control tissues for the anti‐NKCC1 and anti‐KCC2 antibodies were used to confirm the labeling accuracy of these antibodies. The anti‐NKCC1 antibody effectively labeled olfactory sensory neurons (not shown), and adult newborn granule cells (Spampanato et al. 2012), but as expected, failed to label kidney. Similarly, the anti‐KCC2 antibody labeled pyramidal cells in the hippocampus and cortex, but again, as expected, did not label kidney tissue (data not shown).

Together, these results suggest that as suggested in cortical pyramidal neurons, the depolarizing chloride gradient at AAC synapses may result from the low expression of KCC2 (Szabadics et al. 2006). In addition to chloride ions, GABA_A_ receptors are also permeable to bicarbonate (HCO_3_^‐^) ions (Bormann et al. 1987; Staley et al. 1995). The HCO_3_^−^ reversal potential (E_HCO3−_) is typically much more positive than the resting membrane potential due to the intracellular catalysis of HCO_3_^−^ from CO_2_ by carbonic anhydrase. Repetitive activation of GABAergic synapses results in a change in the GABA reversal potential, due a positive reversal potential set by a combination of Cl^−^ influx and HCO_3_^−^ efflux. This change in the anion reversal potential is likely to be rather slow requiring depletion of intracellular chloride (Staley et al. 1995); however, we tested the role of carbonic anhydrase in maintaining a depolarizing E_HCO3‐_ gradient. Acetazolamide (10 μmol/L), an antagonist of carbonic anhydrase (Supuran et al. 2003), also had no effect on the success rate of fbEPSPs (*n* = 4; Fig. 5A).

## Discussion

GABAergic interneurons form diverse populations of cells that are distinguished by expression of different cytosolic markers, physiological properties, and the targets they innervate (Markram et al. 2004; Ascoli et al. 2008; Klausberger and Somogyi 2008). In the mature nervous system, synaptic release of GABA at interneuron synapses activates anion‐selective ion channels and postsynaptic chloride influx. The overall impact of GABAergic synaptic activity is inhibitory due to a combination of membrane hyperpolarization and shunting inhibition. In recent years however, one type of PV‐positive interneuron, which makes synapses on the axon initial segment (AIS) of pyramidal neurons, has been found to be excitatory, capable of driving postsynaptic pyramidal cells to threshold (Szabadics et al. 2006; Molnar et al. 2008). Moreover, pyramidal neurons receiving these excitatory GABAergic synapses in turn synapse back onto the same interneuron, generating a feedback excitatory circuit (McDonald et al. 2005; Szabadics et al. 2006; Molnar et al. 2008). In the BLA, we have shown that this circuit develops about the same time that GABA is undergoing the switch to inhibitory signaling (Ehrlich et al. 2013), and persists to at least P44. This developmental profile matches the onset of PV expression (Davila et al. 2008), and likely reflects a late developmental stage of parvalbumin interneuron synaptic connectivity in the BLA. PV cells in the cortex undergo a dramatic change in gene expression during this postnatal period (P7–P40), consistent with the developmental and physiological changes that we report (Okaty et al. 2009).

We have shown that in the BLA, these PV‐positive feedback interneurons make synapses onto the AIS as well as the soma of local pyramidal neurons. Moreover, these cells also make GABAergic synapses with other neighboring PV+ interneurons (Woodruff et al. 2006). This variety of postsynaptic domain targeting indicates that these PV‐positive interneurons in the BLA are distinct from the highly specialized AACs, which selectively make synapses onto the pyramidal neuron AIS (Somogyi et al. 1982). While cartridge‐type AIS synapses are present in the BLA (McDonald and Betette 2001; McDonald et al. 2002; Muller et al. 2006; Bienvenu et al. 2012; Veres et al. 2014), feedback PV interneurons often made single *en passant* synapses on neighboring AISs. Such single synaptic contacts made by AACs on to AIS is well documented (Somogyi et al. 1982), and diverse target selection has been previously described for stellate cells that synapse onto the soma, dendrites and AIS of principal cells without cartridge‐type axo‐axonic synapses (Peters and Fairen 1978). Thus, the most striking difference between the feedback cells we describe, and AACs is that they also make synapses with other PV‐positive interneurons (Woodruff et al. 2006). Despite the differing morphology between feedback cells in the BLA and cortex, the underlying circuits share common physiological features. First, as in the cortex, PV interneurons in the BLA with feedback excitation are GABAergic (Szabadics et al. 2006; Woodruff et al. 2006; Molnar et al. 2008). Second, as in the cortex, feedback excitation can be blocked by both GABAergic and glutamatergic antagonists (Szabadics et al. 2006; Woodruff et al. 2006; Molnar et al. 2008). Last, these neurons appear to arise later in development but are present in fully mature animals, and GABAergic axo‐axonic cells have been reported to excite pyramidal cells in human cortex in slices taken from patients aged 18–73 years (Molnar et al. 2008).

The feedback circuit has a very high fidelity, such that action potentials in a single PV‐interneuron activate a time‐locked polysynaptic chain of activity that feeds back on to the same cell with few or no failures. The amplitude of the fbEPSC following single action potentials was very variable but could reach up to 1 nA (Fig. 4). On the basis of the amplitude of the average unitary EPSC evoked at principal cell to stuttering and fast‐spiking PV+ interneuron synapses (71 pA and 156 pA respectively) (Woodruff and Sah 2007b), we estimate that spiking in a single parvalbumin interneuron can synchronize the activity of up to 12 principal neurons. Hippocampal and cortical axo‐axonic cells are known to innervate large numbers of pyramidal neurons (Howard et al. 2005; Taniguchi et al. 2013). Similarly, we find that single PV+ interneurons in the BLA that receive fbEPSCs innervate the AIS of up to 20 pyramidal neurons (mean 6.5) (Fig. 2). The presence of neurons with very large amplitude fbEPSCs suggests that most of the principal cells receiving excitatory AIS synapses would also have to synapse back on to the PV interneuron, suggesting a high level of reciprocal connectivity for the BLA. The high fidelity of axo‐axonic to pyramidal cell transmission is likely due to the location of these synapses on the AIS, which also expresses a high density of low‐threshold voltage dependent sodium channels (Kole and Stuart 2012). As in the hippocampus (Cobb et al. 1995), interneurons in the amygdala are able to synchronize the output of principal cells through GABAergic inhibition (Woodruff and Sah 2007a; Popescu and Pare 2011; Ryan et al. 2012). However, if the feedback circuit functions in vivo as described here, PV+ feedback cells may also generate associated synchronized networks of principal cells through direct excitation.

GABAergic excitation is well documented in the developing central nervous system, and is recapitulated during development of newborn dentate granule cells in the adult (Ben‐Ari et al. 2007; Ge et al. 2007). In mature neurons, GABA is generally thought to be inhibitory. However, the fbEPSC present in BLA interneurons, a glutamatergic event, is blocked by inhibiting GABA_A_ receptors, indicating that the released GABA is excitatory in at least some synapses made by these cells. Why is GABA excitatory at some synapses in the adult BLA? NKCC1 was not detectable in the AIS, and fbEPSCs were not affected by blocking NKCC1. Therefore, the excitatory GABA activity cannot result from chloride loading by NKCC1. Consistent with previous observations (Szabadics et al. 2006), KCC2 expression was also not detectable in the AIS suggesting the potential for passive accumulation of chloride in a subcellular compartment lacking an active exporting mechanism. However, given the proximity of the AIS to the soma it seems unlikely that the AIS could passively maintain a relatively high chloride concentration. Alternatively, it has recently been proposed that while chloride transporters play a role in maintaining chloride homeostasis, local impermeant anions may determine the homeostatic set point for [Cl^−^] (Glykys et al. 2014) (Delpire and Staley 2014). This Gibbs–Donnan effect dictates that compartments with a lower concentration of impermeant anions can accumulate displaced chloride compared to areas with higher impermeant anions allowing for a differential distribution of chloride in subcellular compartments. Glykys et al. have demonstrated that, in slices from animals aged P28–30; neocortical pyramidal cells have a significantly higher chloride concentration in their axons compared to somas or dendrites (Glykys et al. 2014).

In addition to the AIS synapses feedback cells also made somatic synapses. Cytosolic chloride levels have been reported to be widely distributed from close to 0 mmol/L to up to 40 mmol/L in CA1 neurons from animals aged P32–44 (Glykys et al. 2014). While only a small fraction of the total population of cells sampled had high chloride, the divergence of parvalbumin interneuron connectivity on to principal cells in the BLA is very high (Muller et al. 2006). Based on the amplitude of the average unitary EPSC evoked at principal cell to stuttering and fast‐spiking PV+ interneuron synapses mentioned above (Woodruff and Sah 2007b), *only a few pyramidal cells* would have to be excited to account for the *average* feedback EPSCs reported here. Therefore, it remains possible that a small number of principal neurons receiving inputs from feedback interneurons maintain a higher cytosolic chloride.

Finally, we cannot rule out the possibility that the feedback excitation results from damaged cells or cut axons that are produced by the tissue preparation technique. However, in a detailed study, Dzhala et al. directly investigated the effect of cellular damage in acute brain slices compared to intact hippocampi (Dzhala et al. 2012). They found that damage during the cutting procedure led to chloride loading and in slices prepared from immature animals produced excitatory GABA actions. In contrast, they found that this slice‐artifact induced by the cutting procedure was not detectable in tissue from older animals aged P21–28 (Dzhala et al. 2012). That is, older slices do not adopt a detectable GABA induced depolarization or excitation as a result of the cutting procedure. However, it is in slices from older animals where we were most able to find feedback cells. Finally, Dzhala et al. demonstrate that the number of damaged neurons was greatly reduced by cutting slices in a high sucrose solution. We have also prepared slices under these conditions to minimize cellular damage. In high sucrose cutting solution, we found no difference in our ability to find feedback interneurons (unpublished results). We have also perfused animals with cold Ringer with high Mg^++^ and low Ca^++^ to minimize damage and again did not find any differences (unpublished results). On the basis of this empirical evidence, we feel that the “slice artifact/cut‐axon” hypothesis is not consistent with these data.

## Conflict of Interest

None declared.
